# Laparoscopic Sacrocervicopexy Using Ethibond Suture Graft: A Very Economic Yet Effective Fertility Preserving Surgery for Pelvic Organ Prolapse

**DOI:** 10.7759/cureus.33086

**Published:** 2022-12-29

**Authors:** Jagadish C Sharma, Avir Sarkar, Neelima Choudhary, Ramesh Chandra, Anupma Anupma, Geetanjali Munda, Ujjwala Anand, Pragathi Kollabathula, Bhumika Jesingh

**Affiliations:** 1 Obstetrics and Gynecology, Employees' State Insurance Corporation (ESIC) Medical College and Hospital, Faridabad, IND

**Keywords:** female sexual function index, gynecologic endoscopy, pelvic organ prolapse, sacrocervicopexy, laparoscopic technique

## Abstract

Background: The modern era has witnessed a transition to a phase of uterus-preserving surgeries and so holds true for pelvic organ prolapse (POP) surgeries as well. Laparoscopic sacrocervicopexy has become a preferred surgical modality for moderate to severe degrees of POP in most women of the childbearing age group. With the alarming incidences of mesh erosion, synthetic mesh has almost gone off the market. We advocate a very simple and cost-effective technique of laparoscopic sacrocervicopexy using an Ethibond suture graft.

Materials and Methods: It was a pilot prospective observational study over one year. Consecutive consenting women with symptomatic prolapsed uterus Stage-II of the central component of the quantitative POP classification (POP-Q) were recruited. Laparoscopic sacrocervicopexy was performed under general anesthesia using the standard protocols, and patients were prospectively followed for six months after surgery. The duration of surgery and hospital stay were noted. Patient satisfaction was rated using a five-point Likert scale. The vaginal length was measured immediately after and six months post-surgery. Sexual function was assessed using the validated female sexual function index (FSFI) scale six months after sacrocervicopexy.

Results: Out of 28 recruited women, the majority were multiparous, highly qualified, and belonged to the middle socio-economic class. Seven patients had co-morbidity in the form of hypertension (17.8%), diabetes (7.1%), and cardiovascular diseases (7.1%). The mean duration of surgery was 105.8±7.2 minutes in the study population. The mean duration of hospital stay was 2.2±0.6 days. No surgical site infection was noted in any of the cases. Most patients rated “very satisfied” experiences following surgery (67.9%). The mean vaginal length after surgery was 7.6±1.2 centimeters. After a follow-up period of six months, the mean vaginal length was 7.4±0.8 centimeters. The mean FSFI score was 30.8±2.4.

Conclusion: Laparoscopic sacrocervicopexy with Ethibond suture graft is a cost-effective and safe surgical technique for POP in resource-limited settings. It also obviates the additional cost of synthetic mesh and the long-term risks of mesh erosion.

## Introduction

Pelvic organ prolapse (POP) is quite common in the developing world and thereby constitutes a major fraction of gynecological surgeries being performed [1]. Over the past few decades, a plethora of pelvic surgeries has been designed to restore the anatomy of the pelvic floor by preserving the vaginal axis, its length, and function in terms of urological and sexual satisfaction [2]. The modern era has witnessed a transition to a phase of uterus-preserving surgeries and so sacrocervicopexy is a preferred modality for moderate to severe degrees of POP in most women of the childbearing age group. For the treatment of vault prolapse, sacrocolpopexy is already the gold standard procedure [3]. The laparoscopic approach to sacrocervicopexy provides the best-combined outcome of abdominal sacropexy with decreased morbidity and surgical site infections [4]. With the alarming incidences of mesh erosion ranging from 0.8% to 9%, the synthetic mesh has almost gone out of the market for performing sacrocervicopexy [5,6]. Moreover, it was quite expensive for patients in low middle income countries (LMIC). This has resulted in the innovation of our present surgical technique.

## Materials and methods

It was a pilot prospective observational study over one year consisting of women deserving fertility preservation surgery for POP. Informed consent was obtained from all participants. All procedures in this study involving human participants were performed in accordance with the ethical standards of the Institutional Ethics Committee of ESIC Medical College and Hospital Faridabad (approval number 134X/11/13/2022-IEC/38) and with the 1964 Helsinki Declaration and its later amendments or comparable ethical standards. Consecutive consenting women with prolapsed uteri during the study time frame of one year with the following inclusion criteria were recruited: age between 35 and 60 years at the time of recruitment, sexually active, symptomatic prolapse, and Stage-II of the central component of the quantitative POP classification (POP-Q) with or without anterior or posterior compartment prolapse, normal Papanicolaou test and fit for undergoing laparoscopic surgery. We excluded patients who were pregnant at the time of recruitment, had concomitant stress urinary incontinence along with POP, were diagnosed with active genital tract infection, or had a history of any chronic systemic illness not fit for undergoing major surgery under general anesthesia.

Participants were explained the procedure in detail. All surgeries were performed by a single surgeon expertized in the technique to avoid any bias. Preoperative skin cleansing with betadine scrub bath one night prior and surgical site application of chlorhexidine was strictly ensured. One dose of first-generation injectable cephalosporin (cephazolin) was administered 60 minutes before the anticipated skin incision. General anesthesia was administered to all patients, and the procedure was performed in semi-lithotomy with the Trendelenburg position allowing both vaginal and laparoscopic access. The urinary bladder was catheterized, and a uterine manipulator was inserted vaginally for ease of manipulation. Pneumoperitoneum was created with carbon dioxide using a Veress needle. A central camera port of 10 millimeters was inserted through the umbilicus, and three five-millimeter trocars were placed laterally on either side lateral to the inferior epigastric vessels for the introduction of the working instruments.

A long graft with a terminal large pore knot was prepared aseptically with Ethibond suture number five. Ethibond excel suture is a non-absorbable, braided surgical suture composed of polyethylene terephthalate (PET) prepared from high molecular weight fibers of long-chain linear polyesters. The highly adherent coating of polybutylate makes the suture nonabsorbable and improves the handling qualities in contrast to other braided uncoated sutures. It is routinely used in orthopedic surgeries for the repair of tendons, ligaments, and joint capsules and for maintaining tension bands in certain fractures.

During laparoscopy, the presacral space was identified. The adjacent vital structures, including the middle sacral and common iliac vessels, the ureter, and the presacral nerve plexus, were delineated. Keeping the right adnexa away from the surgical field, the peritoneum over the sacral promontory was elevated and incised. The anterior longitudinal ligament was cleared off its surrounding loose fibrofatty tissues. This was followed by the creation of a longitudinal space medial to the right uterosacral ligament from the sacral promontory to the posterior surface of the cervix for housing the graft.

Next, the surgeon opened the uterovesical fold of the peritoneum anteriorly and dissected it carefully to retract the bladder fold downwards. The needle, along with the Ethibond suture graft, was passed from the posterior aspect of the left broad ligament through an avascular window lateral to the uterus. Through the uterovesical space, the needle was passed to the right broad ligament, where it was again pierced through an avascular window to emerge into the posterior aspect. The needle was then passed through the preformed knot (thereby putting a reef knot), anchoring the graft around the cervix loosely. The graft was now placed in the space created along the medial aspect of the right uterosacral ligament, and the uterus is elevated to the desired position using the uterine manipulator. A good bite into the anterior longitudinal ligament along the surface of the sacral promontory secures the graft to the bony support. The graft was then covered by re-approximating the cut edges of the retro-peritoneum over the graft.

The duration of surgery was noted. Enhanced recovery after surgery (ERAS) protocol was followed, and patients were discharged early in the postoperative period [7]. The total duration of hospital stay was also noted. Patient satisfaction was rated using a five-point Likert scale (very dissatisfied, dissatisfied, neutral, satisfied, and very satisfied) at the time of discharge from the hospital. The vaginal length was measured immediately after and six months post-surgery using a graduated, pre-marked Ayer’s spatula. At six months follow-up visit, sexual function was assessed using the validated Female Sexual Function Index scale [8]. It is a brief self-reported measure of a woman’s sexual function addressing the multidimensional nature of the sexual function. It consists of six subscales assessing the desire, arousal, lubrication, orgasm, sexual satisfaction, and dyspareunia. A set of 19 questions are distributed in the following domains. The sums of individual domain scores are multiplied by the respective domain factor ratio (0.6 for desire, 0.3 for arousal, 0.3 for lubrication, 0.4 for orgasm, 0.4 for satisfaction, and 0.4 for dyspareunia). The total domain scores are then summed up to derive the total score (ranging from 2.0 to 36.0, worst sexual experience to most pleasurable one).

## Results

A total of 28 women were recruited during the study time frame. The mean age of the participants was 46.6±3.4 years (Table 1). The majority of the patients were multiparous (82.2%), highly qualified (53.6% were graduates and post-graduates), and belonged to the middle socio-economic class (57.2%). Seven patients had co-morbidity in the form of hypertension (17.8%), diabetes (7.1%), and cardiovascular diseases (7.1%). Both patients had mild mitral stenosis and successfully underwent the laparoscopic procedure without any intra-operative complications. The mean duration of surgery was 105.8±7.2 minutes in the study population. Since ERAS protocols were strictly adhered to and patients were encouraged early mobility and discharge in the postoperative period, the mean duration of hospital stay was 2.2±0.6 days. No surgical site infection was noted in any of the cases till now. The majority of the patients rated “very satisfied” following surgery (67.9%). 

**Table 1:** Table showing the results of the prospective observation conducted during the study time frame

S. no.	Characteristics	Parameters	Frequency (n=28)
1.	Mean age of participants (in years)		46.6±3.4
2.	Parity	Primiparous	5 (17.8%)
		Multiparous	23 (82.2%)
3.	Education	Up-to High school	6 (21.4%)
		Higher secondary	7 (25.0%)
		Graduate and post-graduate	15 (53.6%)
4.	Socio-economic status	Lower	3 (10.7%)
		Middle	16 (57.2%)
		Upper	9 (32.1%)
5.	Co-morbidities	Hypertension	5 (17.8%)
		Diabetes	2 (7.1%)
		Cardiovascular diseases	2 (7.1%)
6.	Mean duration of surgery (in minutes)		105.8±7.2
7.	Mean duration of hospital stay (in days)		2.2±0.6
8.	Patient satisfaction	Very satisfied	19 (67.9%)
		Satisfied	7 (25.0%)
		Neutral	2 (7.1%)
		Dissatisfied	0 (0.0%)
		Very dissatisfied	0 (0.0%)
9.	Mean vaginal length immediately after surgery (in centimeter)		7.6±1.2
10.	Mean vaginal length six months after surgery (in centimeter)		7.4±0.8
11.	Mean Female Sexual Function Index (FSFI) score		30.8±2.4

The mean vaginal length after surgery was 7.6±1.2 centimeters (Table 1). After a follow-up period of six months, the mean vaginal length was 7.4±0.8 centimeters. The FSFI score was assessed, and the mean score was 30.8±2.4. The majority of the participants did not face any difficulty with respect to sexual satisfaction, orgasm, arousal, lubrication, or desire and did not report having experienced any degree of dyspareunia.

## Discussion

With the increasing desire for fertility preservation among women, sacrocervicopexy is becoming the preferred surgical modality [9]. After the first report of 13 women with symptomatic uterovaginal prolapse being treated by sacrohysteropexy without any complication in 2001, surgeons all over the world started taking an interest in this technique [10,11]. Another retrospective study of 40 women in 2008 successfully evaluated improved patient satisfaction and less post-operative morbidity in women undergoing sacrohysteropexy [12]. A Cochrane review had shown that the risk of cervical stump carcinoma among women with previously normal Papanicolaou test was approximately 0.3% which was similar to the risk of vaginal cancer following hysterectomy for benign pathologies [13].

Our technique of sacrocervicopexy is simple, economical, and has a short learning curve. Preservation of the cervix avoids the opening of the vagina. Holding the cervix all around with a reef knot has no chance of cervical erosion, tissue remodeling, and loosening of the graft in contrast to the traditional technique of fixing the graft on the posterior surface of the cervix. Suturing is done only once over the anterior longitudinal ligament. No need for multiple sutures for fixing the graft. Thus, this technique is easy for beginners and very economical in minimal resource settings [5,6,9]. Moreover, there is practically no chance of mesh erosion and the need for repeat surgical intervention [10,11]. The preservation of uterosacral ligaments seems to improve the sexual quality of life [4,13].

The added advantages of shorter hospital stays, lower post-operative pain, and rapid return to normal routine activities have led to greater in towards laparoscopic access [4,14]. Laparoscopy also provides a magnification of the surgical field, thereby allowing better and more precise placement of the stitches [14]. The aesthetically appealing look has increased the patient satisfaction scores in our results also. Achieving a significant vaginal length for satisfactory coitus in the immediate post-operative and follow-up periods reiterates the success of this surgical technique. Long-term follow-up of this cohort will give us an idea of the long-term outcome of the procedure.

The modern era has witnessed a paradigm shift of preference towards a desire for uterus preservation surgeries, which, in turn, has led to the innovation of various minimally invasive surgical modalities [15,16]. Ours is an inexpensive and easy technique with good patient satisfaction outcomes to date. The major limitations of this study were: it was a non-randomized single-center study, no comparison was done with other standard surgical techniques, and long-term sexual outcomes are yet to be followed up.

## Conclusions

Laparoscopic sacrocervicopexy with Ethibond suture graft is a cost-effective and safe surgical technique for POP in resource-limited settings of LMIC. It obviates the additional cost of synthetic mesh and the long-term risks of mesh erosion. It does not require a very high degree of laparoscopic suturing skill. High patient satisfaction and ease of surgery have promoted us to adopt this technique in low-resource hospitals.
